# Agro-Physiologic Responses and Stress-Related Gene Expression of Four Doubled Haploid Wheat Lines under Salinity Stress Conditions

**DOI:** 10.3390/biology10010056

**Published:** 2021-01-14

**Authors:** Ibrahim Al-Ashkar, Walid Ben Romdhane, Rania A. El-Said, Abdelhalim Ghazy, Kotb Attia, Abdullah Al-Doss

**Affiliations:** 1 Plant Production, College of Food and Agriculture Sciences, King Saud University, Riyadh 11451, Saudi Arabia; wromdhane@ksu.edu.sa (W.B.R.); aaldoss@ksu.edu.sa (A.A.-D.); 2 Agronomy Department, Faculty of Agriculture, Al-Azhar University, Cairo 11651, Egypt; 3 Biological and Ecological Department, Faculty of Home Economic, Al-Azhar University, Tanta 32514, Egypt; RaniaEl_said@azhar.edu.eg; 4 Center of Excellence in Biotechnology Research, King Saud University, P.O. Box 2455, Riyadh 11451, Saudi Arabia; kattia1.c@ksu.edu.sa; 5 Rice Biotechnology Lab, Rice Research & Training Center, Field Crops Research Institute, Sakha, Kafr EL-Sheikh 33516, Egypt

**Keywords:** wheat, doubled haploid lines, salt tolerance, agro-physiologic traits, abiotic stress-related genes, multivariate analyses

## Abstract

**Simple Summary:**

Productivity of wheat can be enhanced using salt-tolerant genotypes. However, the assessment of salt tolerance potential in wheat through agro-physiological traits and stress-related gene expression analysis could potentially minimize the cost of breeding programs and be a powerful way for the selection of the most salt-tolerant genotype. The study evaluated the salt tolerance potential of four doubled haploid lines of wheat and compared them with the check cultivar Sakha-93 using an extensive set of agro-physiologic parameters and salt-stress-related gene expressions. The results indicated that the five genotypes tested displayed reduction in all traits evaluated except the canopy temperature and electrical conductivity, which had the greatest decline occurring in the check cultivar and the least decline in DHL2. The genotypes DHL21 and DHL5 exhibited increased expression rate of salt-stress-related genes under salt stress conditions. The multiple linear regression model and path coefficient analysis showed a coefficient of determination of 0.93. Concluding, the number of spikelets, and/or number of kernels were identified to be unbiased traits for assessing wheat DHLs under salinity conditions, given their contribution and direct impact on the grain yield. Moreover, the two most salt-tolerant genotypes DHL2 and DHL21 can be useful as genetic resources for future breeding programs.

**Abstract:**

Salinity majorly hinders horizontal and vertical expansion in worldwide wheat production. Productivity can be enhanced using salt-tolerant wheat genotypes. However, the assessment of salt tolerance potential in bread wheat doubled haploid lines (DHL) through agro-physiological traits and stress-related gene expression analysis could potentially minimize the cost of breeding programs and be a powerful way for the selection of the most salt-tolerant genotype. We used an extensive set of agro-physiologic parameters and salt-stress-related gene expressions. Multivariate analysis was used to detect phenotypic and genetic variations of wheat genotypes more closely under salinity stress, and we analyzed how these strategies effectively balance each other. Four doubled haploid lines (DHLs) and the check cultivar (Sakha93) were evaluated in two salinity levels (without and 150 mM NaCl) until harvest. The five genotypes showed reduced growth under 150 mM NaCl; however, the check cultivar (Sakha93) died at the beginning of the flowering stage. Salt stress induced reduction traits, except the canopy temperature and initial electrical conductivity, which was found in each of the five genotypes, with the greatest decline occurring in the check cultivar (Sakha-93) and the least decline in DHL2. The genotypes DHL21 and DHL5 exhibited increased expression rate of salt-stress-related genes (TaNHX1, TaHKT1, and TaCAT1) compared with DHL2 and Sakha93 under salt stress conditions. Principle component analysis detection of the first two components explains 70.78% of the overall variation of all traits (28 out of 32 traits). A multiple linear regression model and path coefficient analysis showed a coefficient of determination (R^2^) of 0.93. The models identified two interpretive variables, number of spikelets, and/or number of kernels, which can be unbiased traits for assessing wheat DHLs under salinity stress conditions, given their contribution and direct impact on the grain yield.

## 1. Introduction

Wheat (*Triticum aestivum* L.) is considered one of the most important cereal grain crops worldwide. It is the dominant source of calories and protein for most countries, giving it an important role in food security. The continuously increasing human population creates a need for continuous increases in grain yield, resulting in serious challenges for plant breeders to generate more varieties with higher productivity [1,2]. Increasing grain yield and tolerance to biotic and abiotic stress conditions are fundamental goals for the genetic improvement of wheat [3]. Efforts are needed to increase yields by selecting traits for higher productivity under abiotic stress conditions, which affect approximately 40–50% of the global land area [4]. Salinity is a serious abiotic stressor and a great concern for plant breeders and researchers in the field of plant physiology and biotechnology, prompting them to reach a credible screening criteria of salt tolerance in wheat genotypes [5]. This aim may be achieved by closely using a combination of classical and modern breeding approaches and selecting reliable phenotypic traits and high computer-powered modeling of multi-trait data to provide a greater understanding of the complicated metabolic mechanisms that function under abiotic stress conditions [1,6,7,8].

Salinity is one of the key factors in climate change. The combination of increasing salinity levels in soil and irrigation water leads to salinity stress, thereby negatively affecting crop performance [8,9]. Stress response in plants is a complex phenomenon that involves a cascade of reactions and the coordinated action of many enzymes and molecules, such as proteins or signaling compounds [10,11]. A better knowledge of the molecular, biochemical, and physiologic mechanisms of salt tolerance will facilitate the screening of genetic material intended for selection, development, and production programs [12,13,14].

Under salt stress conditions, plants suffer from hyperosmotic stress, ion poisoning, and oxidative damage [14]. Salt stress increases the levels of sodium (Na^+^) and decreases potassium (K^+^) concentrations in plant tissues, which are harmful to the plants [15]. A high concentration of Na^+^ inhibits the absorption of essential macro-elements, including K^+^ and calcium (Ca^2+^), from the rhizosphere [16,17]. Subsequently, maintaining a sufficiently high K^+^/Na^+^ ratio remains a challenge for plants that are exposed to salinity stress conditions.

Active transport of Na^+^ to the extracellular space and compartmentalization of Na^+^ to vacuoles are among the best-characterized tolerance mechanisms at the cellular level to limit Na^+^ accumulation in the cytoplasm [15,18]. Membrane transporters, including salt overly sensitive 1 (SOS1), high-affinity K^+^ transporters (HKTs), and Na^+^ proton exchangers (NHXs) are important Na^+^ carrier proteins that cooperatively work to reduce salt toxicity at both the cellular level and the whole-plant level [18,19]. The HKT1 and HKT2 subfamilies of the HKT transporter family are the main players in Na^+^/K^+^ transport at the plasma membrane [15,20]. HKT1 transporters provide selective Na^+^ transport, whereas HKT2 transporters are permeable to both Na^+^ and K^+^ [21]. The transporter TaHKT1;5 was previously confirmed to play an important role in limiting Na^+^ transport from the roots to the leaves in wheat, and RNA interference decreased the levels of TaHKT1;5 gene expression in transgenic wheat lines associated with massive Na^+^ accumulation in the leaves under salt stress conditions [20]. Furthermore, Byrt, et al. [22] reported that downregulation of TaHKT2 gene expression was associated with enhanced salt tolerance in salt-tolerant wheat genotypes. To minimize Na^+^ accumulation in the cell cytosol, plants use Na^+^ exclusion and vacuolar compartmentalization as a tolerance mechanism. This maintains ionic homeostasis in plants subjected to salinity stress [16,23]. SOS1- and NHX-type Na^+^/H^+^ antiporters are the main actors in Na^+^ exclusion and vacuolar compartmentalization [18]. Recently, Almeida, et al. [18] reported that salt-tolerant wheat genotypes upregulated the gene expression of both SOS1 and NHX1 that are associated with low Na^+^ content and low Na^+^/K^+^ ratio.

During salt stress, reactive oxygen species (ROS) generation and accumulation induce chlorophyll degradation and membrane lipid peroxidation [24,25,26]. To eliminate or reduce the deleterious effects of ROS, plants have developed several protective mechanisms that include antioxidant enzymes—such as superoxide dismutase 1 (SOD1), catalase (CAT), glutathione peroxidase 2 (GPX2), and ascorbate peroxidase 1 (APX1)—and non-enzymatic antioxidants [27,28,29]. Several previous studies have reported that salt-tolerant wheat genotypes exhibited increased antioxidant enzyme activities associated with the upregulated expression of their encoding genes, resulting in decreased oxidative damage to these wheat genotypes [26,30,31,32].

It was found that canopy temperature (CT) is a useful indicator of water status in plants [33], which is a tool for the indirect selection of genotypes that are tolerant to stressful environments, and has been proven to be correlated with different morpho-physiologic and agronomic parameters under stress conditions, such as photosynthetic capacity, chlorophyll content, grain yield, and different traits related to water status in plants [34,35,36]. In plant breeding programs, the genotypes that maintain lower CT, transpiration, and gas exchange are more desirable compared with other genotypes under the same experimental conditions [37,38].

Generally, most agronomic traits display polygenic inheritance (indicating that they are regulated by multiple genes) and are highly affected by the environment [39]. Thus, screening of plant salt stress responses in an open field setting may have low accuracy due to complex interactions across genotypes and the environment as well as due to the variability within plants affected by salinity [12]. Selection of breeding lines with the capability to increase yield and enhance salt tolerance in advanced growth media often required repetition for result validation; however, repetition sometimes produces inconsistent results because of the complicated genetic behavior of yield and salinity traits [40]. This methodology is time-consuming and costly, because it requires more than one field assessment, which must be performed during different seasons and locations [12,41]. To simplify the difficult process of selection, using rapid, convenient, and cost-neutral methods that help breeders in assessing key plant traits relevant to stress tolerance may be necessary. Therefore, assessing a plant’s potential to cope with salt stress may be more precise when conducted under controlled conditions [42,43].

Breeding strategies to develop salt-tolerant genotypes depend on assessment of several genotypes (varieties or lines) using multidimensional approaches of phenotypic variations to numerous traits. These integrative approaches combine various parameters of physiologic traits at the leaf and root or whole-plant level, agronomic traits, and molecular characteristics [5,27,44] to understand the mechanisms behind the adaptive behaviors of plants toward stress conditions and to make available comprehensive information to plant breeders regarding the reliability of several components of plant measurements as screening criteria for salinity tolerance as well as the potential for integrating these measurements into breeding programs [5,45,46]. Large datasets from screening tests demand appropriate statistical analysis to elaborate conclusions on tolerant and sensitive genotypes. Therefore, several studies have focused on multivariate analysis techniques, including multicollinearity, multiple regression, principal component, and path analyses, as useful tools for identifying sources of variation to salt tolerance using accurate and multiple selection criteria [44,47,48,49].

In the present study, we evaluated the salt tolerance potential of four doubled haploid lines (DHLs) of bread wheat and compared them with the check cultivar Sakha-93 (salt-tolerant); these wheat varieties were subjected to 150 mM NaCl under greenhouse conditions. Therefore, the present study aims to understand how these tolerance mechanisms work based on agro-physiologic responses and stress-related gene expression and how these strategies effectively balance each other, which may potentially minimize the cost of breeding programs that pursue salt-tolerant varieties. Our goal was also to select the most salt-tolerant genotype to serve as a useful genetic resource for future breeding programs.

## 2. Materials and Methods

### 2.1. Plant Materials

The present study used five wheat genotypes to measure sustainability under salinity stress conditions. We selected four DHLs based on their performance for grain yield under normal conditions [50] and salinity tolerance in the seedling stage at 28 d [5] and in the vegetative stage at 43 d [8]. The DHLs—DHL2, DHL5, DHL21, and DHL26 were obtained from the Agronomy Department, Faculty of Agriculture, Al-Azhar University, Nasr City, Cairo, Egypt [50], whereas grains of salt-tolerant check cultivar (Sakha-93) were provided by the Agricultural Research Center, Egypt. The five genotypes were grown in a greenhouse at the Plant Production Department, College of Food and Agriculture Sciences, King Saud University.

### 2.2. Growth Conditions, Salinity Treatments and Experimental Design

The grains of the five genotypes were incubated at 10 °C for 3 d to eliminate any grain dormancy. Under greenhouse conditions, the experiments were performed at 25 °C–30 °C during the day and 18 °C–20 °C during the night, with a 12-h light/dark photoperiod cycle, and a light intensity of nearly 60 μmol m^−2^ s^−1^. We planted five grains from each genotype in plastic pots (dimensions—22.5 cm diameter at the rim and 19 cm height) filled with sand and peat (2:1). Then, we irrigated the pots with 500 mL of 25% Hoagland nutrient solution twice a week [51]. After two weeks, 150 mM NaCl was added to the nutrient solution until maturity (to avoid osmotic shock, NaCl was progressively applied for 15 d). The control plants were watered in the same way but without NaCl. Treatments (30 experimental units per salinity level) were performed in a randomized complete block factorial design. The average value of three samples and/or plants per replicate (pot) and triplicate per all treatments (genotypes and salinity levels) were used to study the indicated traits.

### 2.3. Measurements

#### 2.3.1. Leaf Water Status Parameters

Leaf water status was measured through three parameters: CT, Leaf water content (LWC), and relative water content (RWC) of the flag leaf at the floret initiation stage. CT was measured under a cloudless sky before noon (11:30 am–12:00 pm) using an infrared thermometer (Therma CAM SC 3000 infrared camera, FLIR System Inc., North Billerica, MA, USA). To measure LWC and RWC, approximately 5-cm-long leaf samples were excised and directly weighed to determine fresh weight (FW). Then, the same samples were soaked in distilled water at 25 °C until they were entirely turgid, carefully drained, placed between tissue paper to remove the remaining water on the leaf surface, and weighed to determine turgid weight (TW). Finally, the leaf samples were oven-dried at 70 °C for 48 h and weighed to obtain dry weight (DW). FW, TW, and DW values were used to calculate LWC and RW as previously described by Clarke and McCaig and Barrs [52,53], respectively, in accordance with the following formulas:

(1) LWC = FW − DW/FW × 100

(2) RWC = FW − DW/TW – DW × 100

#### 2.3.2. Chlorophyll and Leaf Pigment Content Parameters

Leaf chlorophyll content was measured through spectrophotometric analysis of chemically excavated pigments as previously described by by Armon [54]. For each treatment, three leaf disc tissues with a diameter of 7 mm (area = 0.38 cm^2^) were collected from flag leaves in separate Eppendorf tubes and added with 2 mL of 80% acetone at 4 °C for 48 h. Chlorophyll content (a, b, and total; t) were determined through the absorption of the leaf extract at 645 and 663 nm in a double-beam UV-V is spectrophotometer (Ultrospec 2100 Pro, Holliston, MA, USA). Pigment contents were counted using the following formulas:

Chlorophyll a (μg) = [(12.7 × OD at 663 nm) − (2.69 × OD at 645 nm)] × 2 mL acetone_80%_/leaf tissue (3)

Chlorophyll b (μg) = [(22.9 × OD at 645 nm) − (4.68 × OD at 663 nm)] × 2 mL acetone_80%_/leaf tissue (4)

Chlorophyll t (μg) = [(20.2 × OD at 645 nm) − (8.02 × OD at 663 nm)] × 2 mL acetone_80%_/leaf tissue (5)

#### 2.3.3. Flag Leaf Parameters

At the flowering stage, we assessed the morphologic traits of flag leaves, such as flag leaf length (FLL; cm), flag leaf width (FLW; cm), flag leaf area (FLA), and flag leaf angle (FL angle). Using a ruler, FLL was measured as the length from the base to the tip of the flag leaf. Additionally, FLW was measured at the widest part of the leaf. The derived trait, FLA (FLL × FLW × 0.83), was also calculated. FL angle was measured as the angle between the stem immediately below the spike and the flag leaf midrib, with more upright leaves having a smaller leaf angle.

#### 2.3.4. Membrane Injury Parameters

For membrane injury, we collected leaf samples (approximately 5 cm long) of flag leaf at the floret initiation stage and incubated them in tubes with 25 mL of distilled water. Samples were retained for 24 h, and then initial electrical conductivity (EC initial) was measured using a conductivity meter (Oakton PC2700, Vernon Hills, IL, USA). After that, final conductance (EC final) was obtained from the same leaf samples, specifically after autoclaving at 100 °C for 15 min and after cooling to room temperature. Membrane stability index (MSI) and leakage index (the relative loss of intracellular electrolytes from used leaf tissue) were calculated using the following formulas proposed by Sairam et al. [34] and Clarke and Grzesiak et al. [43], respectively:

(6) $$\mathrm{MSI}\;=\;\left[1\;-\mathrm{EC}\;\mathrm{initial}/\mathrm{EC}\;\mathrm{final}\right]/100$$

(7) $$\mathrm{LI}\;=\;\left[S\;\left(\mathrm{EC}\;\mathrm{initial}/\mathrm{EC}\;\mathrm{final}\right)\;-\;C\;\left(\mathrm{EC}\;\mathrm{initial}/\mathrm{EC}\;\mathrm{final}\right)\right]/C\left(\mathrm{MSI}\right)\;\times \;100$$

where, C and S refer to the conductivity of the control (C) and salinity (S) treatment groups, respectively.

#### 2.3.5. Photosynthetic Parameters

Photosynthetic parameters were measured using flag leaves at the floret initiation stage between 10:00 am and 11:00 am. Leaf gas exchange was measured using a Li-6400 gas exchange system (Li-Cor, Inc., Lincoln, NE, USA) to obtain the values for the following parameters: photosynthesis rate (Pn), transpiration rate (E), stomatal conductance (Gs), and intracellular CO_2_ concentration (Ci). All parameters were taken at a photosynthetic photon flux density of 700 μmol photons m^−2^ s^−1^. Temperature was at 26 °C ± 2 °C, CO_2_ concentration was at 485 ± 23 μmol/L, and relative humidity was at 65% ± 7%.

#### 2.3.6. Ion Concentrations

Oven-dried samples of whole shoots, leaf, stem, and spike components were collected after harvesting and ground to a fine powder. The samples were digested, and 0.4 g of each sample was taken and soaked in 8 mL of concentrated HNO3 and 3 mL of HClO_4_ for 12 h and burned at 300 °C for 3 h. Then, we added distilled water to the digested samples up to a final volume of 50 mL. Na^+^ and K^+^ concentrations were measured using a flame photometer (ELEX 6361, Eppendorf AG, Hamburg, Germany), and K+/Na^+^ ratio was determined.

#### 2.3.7. Yield and Yield Components Parameters

Upon reaching maturity, the plants (110–123-day-old) were harvested and threshed to measure the following parameters: spike length (SL; cm) after excluding awns, number of spikelets (NSS; spike^−1^), number of kernels (NKS; spike^−1^), 100-kernel weight (100-KW; g), total grain yield (GY; plant^−1^), and phenotypic data for five-kernel size. Details about five-kernel size traits, that is, kernel length (KL; mm), kernel width (KW; mm), kernel area (KA; mm^2^), and kernel length–width ratio (KLWR), were calculated from 10 kernels per replicate across all treatment groups. KA was calculated using the formula KL × KW × 0.745. KLWR was calculated by dividing the kernel length mean by the KW mean per replicate.

### 2.4. Stress-Related Gene Expression Profiling

Total RNA was extracted from the leaves of two-month-old wheat plants (0.1 g) of each genotype using the method previously described by Ben-Romdhane et al. [55]. RNA samples (3.0 μg) that were previously treated with DNase I (Promega, Madison, WI, USA) were used for cDNA synthesis using the Omniscript RT Kit (QIAGEN GmbH, Hilden, Germany). Real-time quantitative PCR (qRT-PCR) was performed using an ABI 7500 machine (Applied Biosystems, Waltham, MA, USA) with Luna Universal qPCR Master Mix (NEB) according to the manufacturer’s instruction. The PCR cycle conditions were according to those previously described by Ben-Romdhane et al. [55]. The Primer Quest program was used to design the primer pairs of the stress-related genes and the internal reference actin gene. Primer pair sequences are listed in Table S1. The relative expression levels of *T. aestivum* catalase gene (TaCAT1, GU984379), *T. aestivum* Na^+^/H^+^ antiporter gene (TaNHX1, AY040245.1), and *T. aestivum* high-affinity potassium uptake transporter gene (TaHKT1;5, U16709.1) were estimated from triplicate measurements using RT-qPCR, and melting curve analysis was performed to monitor the specificity of the primers. Actin gene (TaAct, AB181991), a housekeeping gene, was used as the internal control. Quantification of relative stress-related gene expression was performed using the comparative CT method [56].

### 2.5. Statistical Analysis

Analysis of variance of different measured traits were performed based on a randomized complete block factorial design (two-way ANOVA) to test the effects of each level of salinity and genotypes and the interactions among them. The F-values of the ANOVA test were calculated for all measured traits. The means performance for the interaction between salinity levels and genotypes were compared using Duncan’s multiple range test at a 95% level of probability. Multicollinearity analysis was used to identify the source and gravity of the multicollinearity in the correlation matrix of interpretive traits to their exclusion. All measured traits for all genotypes across two levels of salinity were analyzed using principal component analysis (PCA) to better evaluate the mutual relations between different measured traits, classify the contributions of these traits, and classify numerous variables into major components. PCA was performed based on the data of the correlation matrix to reduce the dimensions of the data space. The first two PCA components explained the variability in the data. The different interpretive traits were further analyzed using multiple linear regression (MLR) methods to identify the most influential interpretive traits accounting for the most variability in dependent traits. For each method, only influential and significant interpretive traits (*p* ≤ 0.05) were maintained in the models. Finally, to separate the value of the coefficient of determination (R^2^) into direct and indirect effects using path analysis, we considered the outcomes of the MLR methods as interpretive variables for the dependent variable (yield). All statistical analyses were performed using the XLSTAT statistical package software (ver. 2019.1, Excel Add-ins soft SARL, New York, NY, USA).

## 3. Results

### 3.1. Comparative Performance of Agro-Physiologic Parameters

#### 3.1.1. Leaf Water Status Parameters

**Figure 1:**
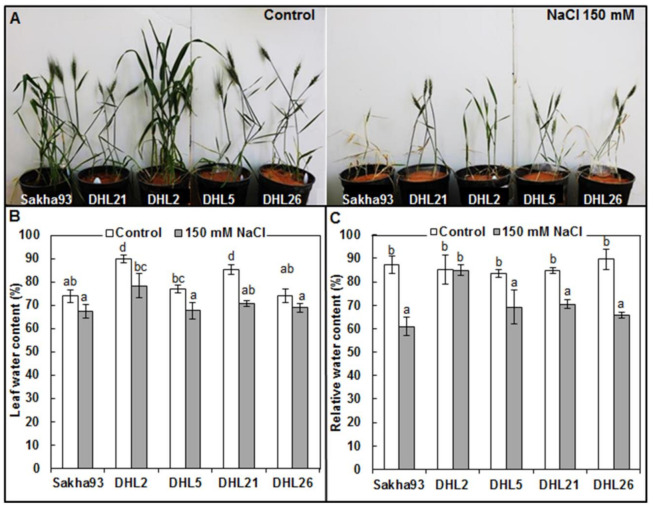
Assessment of salt stress tolerance of five wheat genotypes. (**A**) Phenotype of the five wheat genotypes at the floret initiation stage grown under normal and salt stress conditions. (**B**) Leaf water content. (**C**) Relative water content. Values are presented as the mean ± SE of at least three replications, and values sharing the same letter for each treatment × genotype combination are not significantly different (*p* ≤ 0.05) according to Duncan’s multiple range test.

The leaf water status measurements (LWC, RWC, and CT) showed significant differences (*p* < 0.01) between salinity treatments, genotypes, and their interactions. As shown in Figure 1B, LWC and RWC significantly decreased under salt stress conditions. LWC reduced from 89.91% to 78.34%, from 76.76% to 67.65%, and from 85.31% to 70.59% in DHL2, DHL5, and DHL21, respectively; in exchange, there was no significant reduction in LWC in either DHL21 or Sakha93. For RWC, a significant reduction in four genotypes (DHL5, Sakha93, DHL21, and DHL26) was observed. DHL2 did not show any significant loss in RWC. In contrast, all genotypes showed a significant increase in CT under salinity condition (Figure S1). The increase in CT ranged from 22.33 °C (stress) for DHL21 to 24.36 °C (stress) for Sakha93, which were 21.33 °C and 21.55 °C for the control, respectively.

#### 3.1.2. Chlorophyll and Leaf Pigment Content Parameters

**Figure 2:**
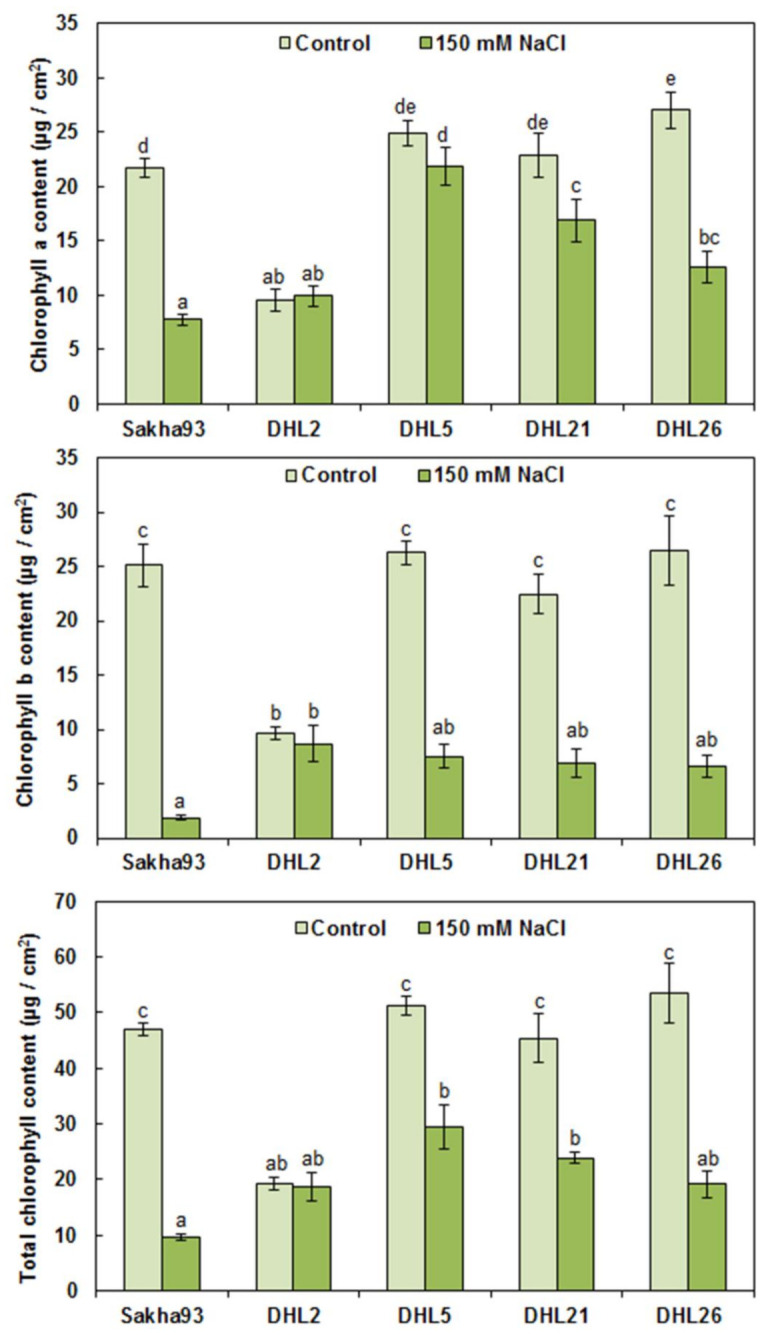
Effects of salinity on chlorophyll a, chlorophyll b, and total chlorophyll content of leaves of wheat genotypes. Values are presented as the mean ± SE of at least three replications, and values sharing the same letter for each treatment × genotype combination are not significantly different (*p* ≤ 0.05) according to Duncan’s multiple range test.

**Figure 3:**
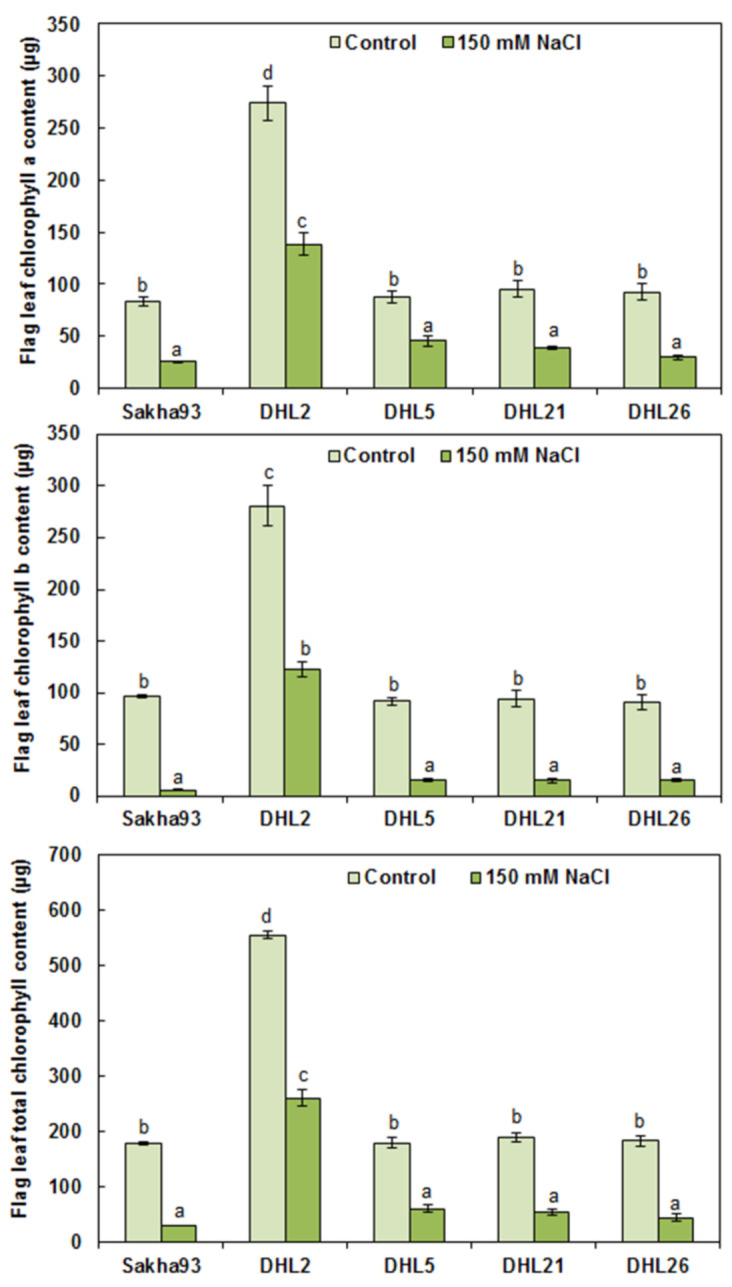
Chlorophyll a, chlorophyll b, and total chlorophyll content of flag leaves of wheat genotypes subjected to irrigation water with or without 150 mM NaCl. Values are presented as the mean ± SE of at least three replications, and values sharing the same letter for each treatment × genotype combination are not significantly different (*p* ≤ 0.05) according to Duncan’s multiple range test.

Considering the results of the ANOVA analysis, Chl content (a, b, and total) in the FLA (0.38 cm^2^) and total pigment content in the flag leaf per plant were significantly different (*p* < 0.001) among the salinity treatments, genotypes, and their interactions (Figure 2 and Figure 3). In general, FLA (0.38 cm^2^) showed a significant decrease in the chlorophyll content (a, b, and total) under salinity stress conditions, compared with control, and a drastic reduction in Chl content in Sakha93 and DHL26 was observed. Interestingly, there was no significant reduction in Chl content in either DHL2 or DHL5 under salt stress conditions, even though DHL2 had a lower Chl content under normal conditions (Figure 2). In contrast, all genotypes showed a significant decrease in total pigment content (a, b, and total) under salinity stress conditions (Figure 3). These decreases (b and total) were up to three-fold, except for DHL2, which reached 50% loss.

#### 3.1.3. Membrane Injury Parameters

**Figure 4:**
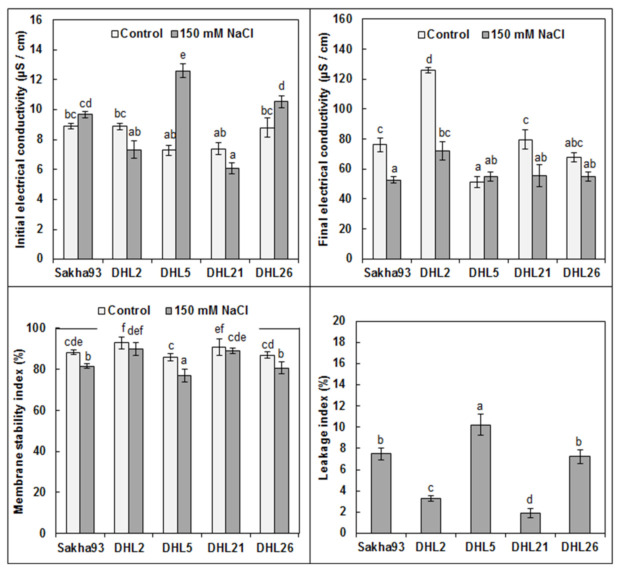
Estimation of membrane injury parameters in five wheat genotypes under normal and salt stress conditions. Values are presented as the mean ± SE of at least three replications, and values sharing the same letter for each treatment × genotype combination are not significantly different (*p* ≤ 0.05) according to Duncan’s multiple range test.

Membrane injury was measured through EC initial, EC final, MSI and LI, which showed significant differences among the studied genotypes (*p* < 0.001) at the floret initiation stage (Figure 4). We observed an increase in EC initial in DHL5 (from 7.28 to 12.60), DHL2 (from 7.33 to 8.87), and DHL26 (from 8.80 to 10.53). Similarly, EC final in Sakha93 (from 52.60 to 76.03) and DHL21 (from 55.70 to 79.53) was increased, whereas a drastic increased in DHL2 (from 71.93 to 125.93) was observed. For MSI, a significant reduction in DHL5 (from 85.83 to 77.02%), Sakha93 (from 88.19 to 81.56%), and DHL26 (from 86.94 to 80.68%) was observed, whereas the decrease in DHL21 and DHL2 was insignificant. Regarding LI, all genotypes showed significant differences among themselves, excluding Sakha93 and DHL26.

#### 3.1.4. Photosynthetic Parameters

**Figure 5:**
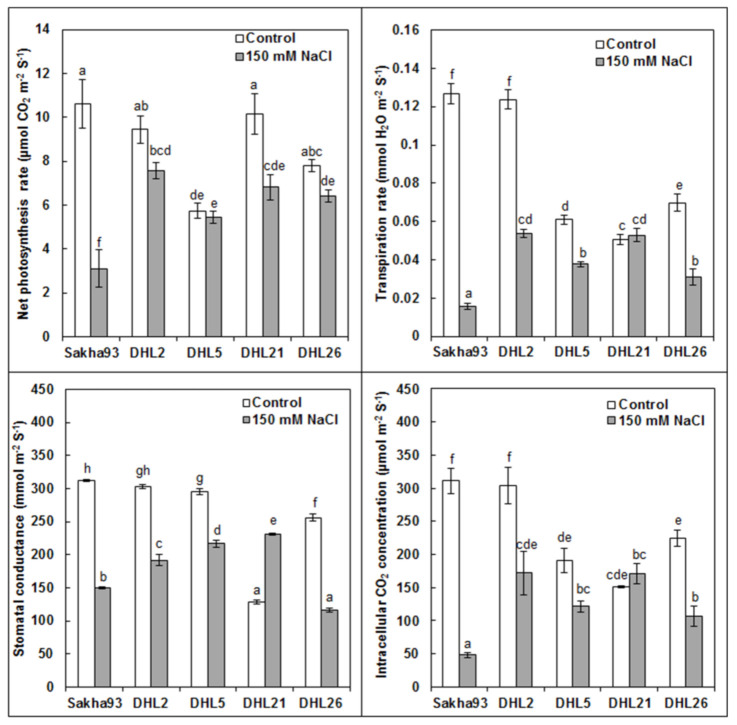
Evaluation of photosynthetic parameters in five wheat genotypes under normal and salt stress conditions. Values are presented as the mean ± SE of at least three replications, and values sharing the same letter for each treatment × genotype combination are not significantly different (*p* ≤ 0.05) according to Duncan’s multiple range test.

Photosynthetic measurements, namely, Pn, Gs, Ci, and E showed significant differences between the treatments (genotypes and salinity levels) and their interactions (*p* < 0.001) at the beginning of the floret initiation stage (Figure 5). Pn had a significantly lower value under salinity conditions, compared with the control in DHL21 and DHL26, whereas a sharp reduction in Sakha93 (from 10.61 to 3.10; more than 70%) was observed. On the contrary, DHL2 and DHL5 showed a small decline, which reflects the relative superiority of these two lines in maintaining chlorophyll pigment safety and photosynthetic rate under salinity stress conditions. Similarly, a significant reduction was observed in Gs in DHL5 and DHL26, whereas a drastic reduction in DHL2 (from 0.12 to 0.05) and Sakha93 (from 0.13 to 0.02) was observed; however, DHL21 showed an insignificant decrease. For Ci, a significant reduction was observed in all genotypes under salinity stress conditions, whereas a lower value was observed in Sakha93 and DHL2, compared with the control. Regarding the value of E, DHL2, DHL5, and DHL26 showed a significantly lower value under salinity stress conditions compared with the control, whereas the decrease in Sakha93 was sharp, and in exchange, the decrease in DHL21 was insignificant.

#### 3.1.5. Flag Leaf Parameters

**Figure 6:**
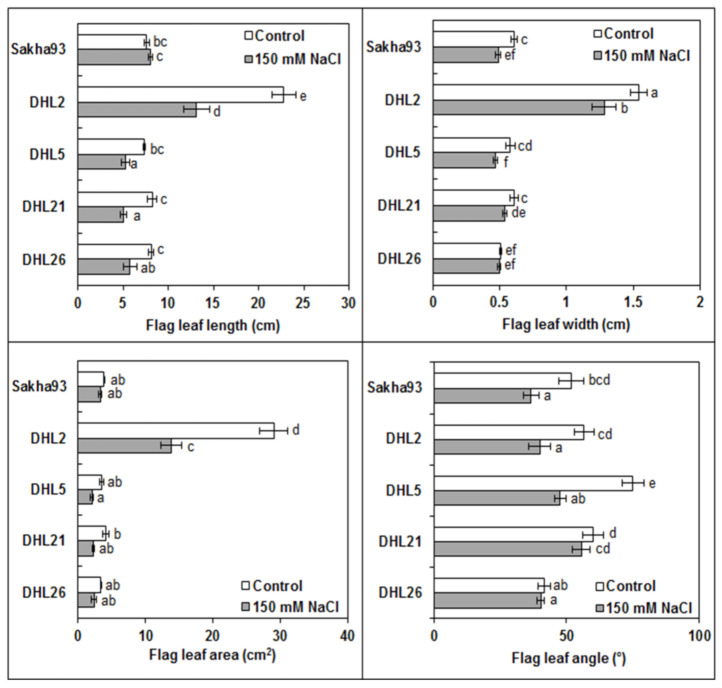
Impact of salinity on flag leaf characteristics in five wheat genotypes subjected to irrigation water with or without 150 mM NaCl. Values are presented as the mean ± SE of at least three replications, and values sharing the same letter for each treatment × genotype combination are not significantly different (*p* ≤ 0.05) according to Duncan’s multiple range test.

The four flag leaf measurements, that is, FL, FW, FLA, and FL angle, showed significant differences (*p* < 0.001) for all treatments (salinity and genotypes) and their interactions (Figure 6 and Figure S2). Flag leaf measurements showed a significant decrease under salinity stress conditions. DHL2 showed a considerable drop in FL (from 22.78 to 13.15, more than 43%) without any significant difference in Sakha93 (Figure 6 and Figure S2). Similarly, a significant reduction in both FW and FL angle in Sakha93, DHL2, and DHL5 was observed; however, DHL21 and DHL26 showed insignificant changes in both FW and FL angle. Actually, none of the three genotypes (Sakha93, DHL5, and DHL26) showed a significant reduction in FLA, but FLA considerably declined in DHL2 (from 29.03 to 12.82, more than 56%) and DHL21 (from 4.15 to 2.28, more than 45%).

#### 3.1.6. Shoot Ion Concentrations

**Figure 7:**
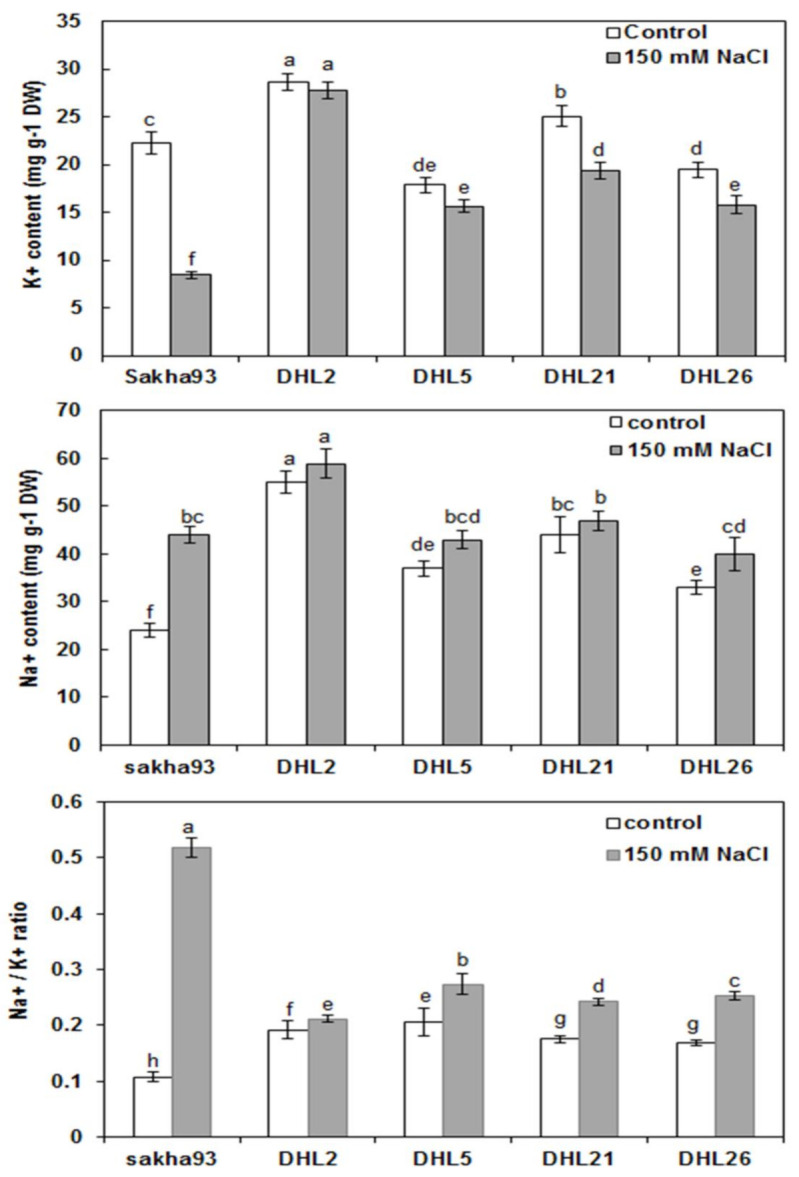
Estimation of Na^+^ and K^+^ accumulation in leaves of five wheat genotypes under normal and salt stress conditions. Values are presented as the mean ± SE of at least three replications, and values sharing the same letter for each treatment × genotype combination are not significantly different (*p* ≤ 0.05) according to Duncan’s multiple range test.

**Figure 8:**
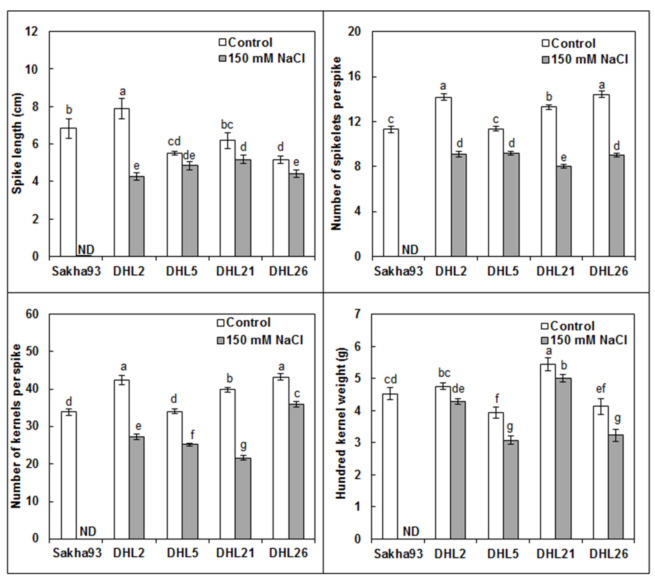
Effects of salinity on yield parameters (spike length, spikelet number, kernel number, and 100-kernel weight) of five wheat genotypes. Values are presented as the mean ± SE of at least three replications, and values sharing the same letter for each treatment × genotype combination are not significantly different (*p* ≤ 0.05) according to Duncan’s multiple range test.

The ANOVA analysis for the Na^+^ and K^+^ ion concentrations and Na^+^/K^+^ ratio showed significant differences (*p* < 0.001) for the salinity levels, genotypes, and their interactions (Figure 7). As shown in Figure 8, K^+^ significantly decreased in Sakha93, DHL21, and DHL26 under salt stress conditions; in exchange, there was no significant reduction in K^+^ in either DHL2 or DHL5. For Na^+^, a significant increase in two genotypes (Sakha93 and DHL26) was observed. DHL2, DHL5, and DHL21 had no significant increase in Na^+^ concentration. Regarding the Na^+^/K^+^ ratio, all genotypes showed a significant increase under salinity stress conditions; however, a drastic increase in Sakha93 (from 0.11 to 0.52, more than 79%) was observed.

#### 3.1.7. Yield and Yield Component Parameters

**Figure 9:**
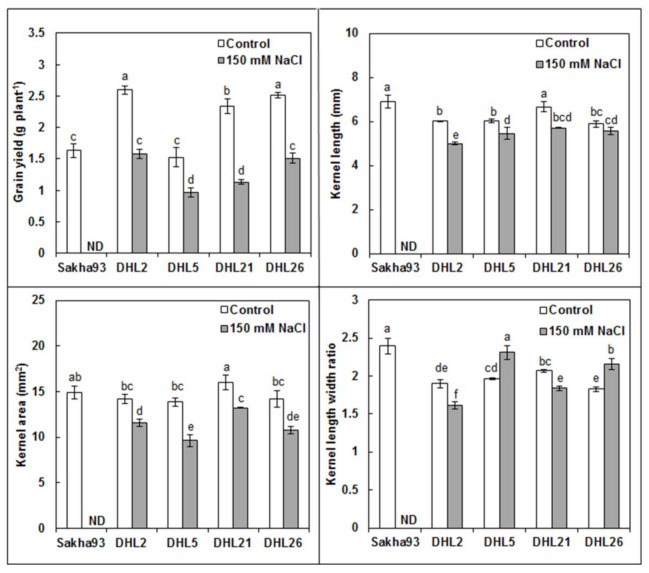
Impacts of salt stress on yield parameters of five wheat genotypes subjected to irrigation water with or without 150 mM NaCl. Values are presented as the mean ± SE of at least three replications, and values sharing the same letter for each treatment × genotype combination are not significantly different (*p* ≤ 0.05) according to Duncan’s multiple range test.

The findings of the ANOVA analysis implied significant differences (*p* < 0.001) for SL, NSS, NKS, HKW, and GY measurements, as well as phenotypic data for four kernel parameters (KL, KW, KA, and KLWR) (Figure 8, Figure 9, Figures S3 and S4) among the nine treatments. Sakha93 showed no life under salinity stress conditions at the beginning of the flowering stage. All yield and yield component measurements showed a significant decrease under salinity stress conditions. The four DHLs showed a significant reduction under salinity stress conditions, relative to control, for all measurements (excluding DHL5 in SL, DHL2 and DHL21 in KW, and DHL26 in KL, wherein the decreases were insignificant). A drastic reduction in DHL2 for SL and DHL21 for NKS was observed. The decrease in GY ranged from 36.70% for DHL5 to 51.51% for DHL21.

### 3.2. Expression Analysis of Related Genes under Salt Stress Conditions

**Figure 10:**
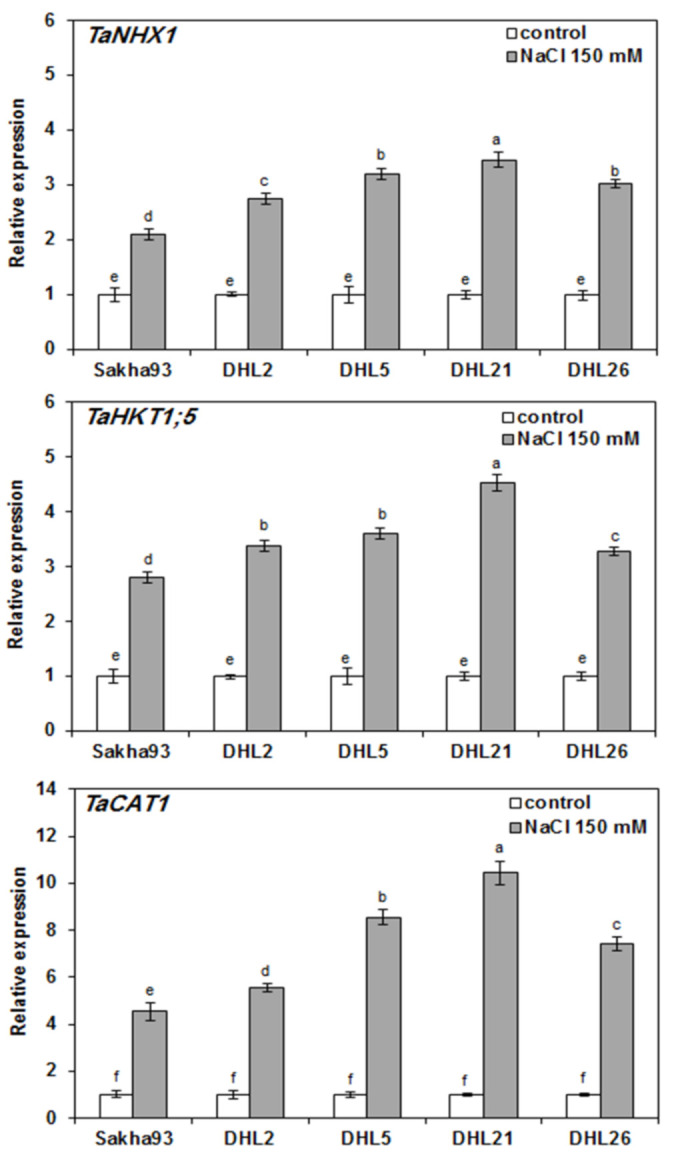
Gene expression profiles of three salt-stress-related genes in leaves of five wheat genotypes subjected to irrigation water with or without 150 mM NaCl. Values are presented as the mean ± SE of at least three replications, and values sharing the same letter for each treatment × genotype combination are not significantly different (*p* ≤ 0.05) according to Duncan’s multiple range test.

Three stress-related genes, TaNHX1, TaHKT1; 5, and TaCAT1 encoding wheat NHX1, HKT1; 5, and CAT1 proteins, respectively, were subjected to qPCR analysis. Compared with normal conditions, the q-PCR analysis demonstrated that the three genes were downregulated in all the genotypes under salt stress conditions, confirming that the chosen genes were salt stress-related. NHX1 transcription rate was strongly upregulated in DHL21, DHL5, DHL26, and DHL2 (3.4-fold, 3.2-fold, 3-fold, and 2.7-fold, respectively), whereas these genes were upregulated to slightly higher levels in Sakha93 under salt stress conditions, compared with normal conditions (Figure 10). Similarly, the expression level of HKT1; 5 gene was higher in DHL21 (4.5-fold), followed DHL5 (3.6-fold), compared with that in other genotypes under normal conditions, whereas the DHL26 and Sakha93 had HKT1; 5 expression levels that were slightly upregulated (3.2-fold and 2.1-fold, respectively). Interestingly, the expressions of NHX1 and HKT1;5 genes were higher in DHL21 and DHL5 compared with DHL26, DHL2, and Sakha93, which showed significantly less upregulation of these genes that are involved in maintaining ion homeostasis. The significant deregulation of these genes in most genotypes confirmed their involvement in salt stress responses. The TaCAT1 gene encodes a catalase protein involved in detoxifying H_2_O_2_ in plants subjected to salinity stress. The expression of this gene was found to be highly upregulated under salt stress conditions, particularly in DHL21 and DHL5, with 2.5-fold and 2-fold increases, respectively, compared with other genotypes that relatively exhibited less upregulation of this gene. Overall, the genotypes that showed a higher expression of these salt-stress-related genes exhibited better tolerance to salinity (Figure 10).

### 3.3. Relationships between Measured Traits and Multivariate Analysis

#### 3.3.1. Relationships between Different Measured Traits

**Figure 11:**
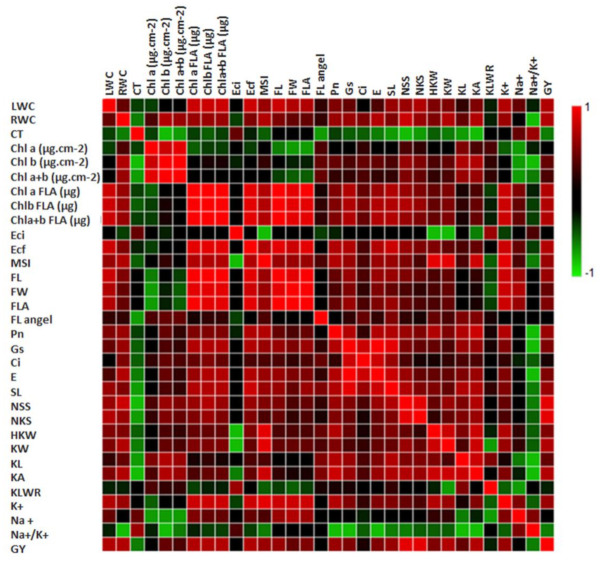
Correlation matrix among 32 measured traits of nine treatments.

To understand the relationships between traits and their contribution to the final product, the multicollinearity between all interpretive traits were analyzed, which implies the lack of any collinearity between traits. Figure 11 shows the correlation matrix between 32 measured traits across the five genotypes and two salinity levels. Generally, the nine measured interpretive traits [LWC, RWC, Chl a (0.38 cm^2^), Chl b (FLA), Chl a + b (FLA), EC final, NSS, NKS, and KA] reflected a positive correlation with GY, which ranged from 0.67 to 0.92. The results reflected positive correlations between all interpretive traits in most cases, except CT, EC initial, KLWR, Na^+^, and Na^+^/K^+^ ratio. NSS showed a significantly positive correlation between Chl b/FLA (*r* = 0.669), NKS (*r* = 0.899), KA (*r* = 0.697), and GY (*r* = 0.924).

#### 3.3.2. PCA of Different Measured Traits

**Figure 12:**
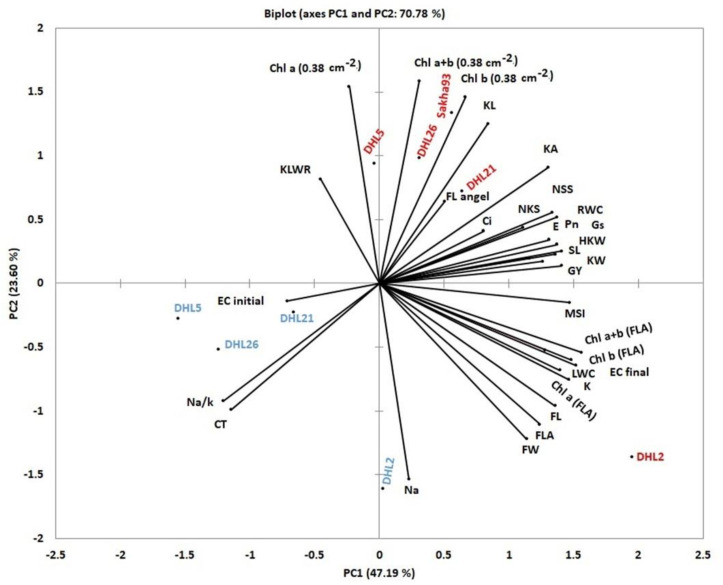
Principal components analysis (based on correlation matrix) of nine treatments.

A PCA was conducted for all measured traits, salinity levels, and DHLs (Figure 12). The PCA resulted in a clear distinction between the two levels of salinity and DHLs due to trait coalitions that identified the main trait, which interpreted much of the variations in the DHLs. The first and second PCA explained 70.78% (47.19% and 23.60%, respectively) of the overall variation of all traits. Furthermore, 28 out of 32 measured traits were loaded onto PC1 and PC2 (with scores > 0.348), and the remaining traits (EC initial, KLWR, Ci, and FL angle) were loaded onto PC3, PC4, and PC6, respectively (Table S2). All measured traits, except five traits, were grouped together in a positive direction onto PC1, and several of them identified with the more tolerant line (DHL2). Three out of five traits were grouped in a negative direction close to DHL5, DHL21, and DHL26 under 150 mM NaCl treatment. Importantly, an acute angle (less than 90°) was formed between the vector of the GY trait and both the measurements of leaf water status (except CT), chlorophyll content (except Chl a (0.38 cm^−2^)), membrane injury (EC initial), photosynthetic, flag leaf, shoot K^+^ ion concentration, and yield components (except KLWR), which signals a positive correlation, whereas the angle between the vector of the GY trait and CT, Chl a (0.38 cm^−2^), EC initial, Na^+^, and Na^+^/K^+^ ratio was greater than 90°, thereby signaling a negative correlation (Figure 12).

#### 3.3.3. Multiple Linear Regression and Path Analysis

Here, 28 out of 32 measured traits were loaded onto PC1 and PC2 and were used to estimate MLR and path coefficient. Table 1 summarizes all the multiple linear regressions that showed significant associations for only four of the influential interpretive traits (MSI, Chl a (0.38 cm^−2^), NSS, and NKS). In the case without deletion, the best model regression showed significant associations with MSI, and NKS contributed toward 0.94; whereas the stepwise and forward models showed significant associations with Chl a (0.38 cm^−2^), and NSS contributed toward 0.08 and 0.85, respectively, with an R^2^ of 0.93 of each of them. After the deletion of NKS, the three models showed significant associations with Chl a (0.38 cm^−2^), and NSS contributed toward 0.93 of each of them. In the case of NSS deletion, the three models of regression showed significant associations with MSI, and NKS contributed toward 0.94 of each of them. Path coefficient analysis separated the R^2^ values into direct and indirect effects (Table 1). The three models of regression showed the importance of NSS or NKS, which had very high direct effects contributing to GY. The R^2^ values were 0.82 and 0.85 for NKS and NSS, respectively. Thus, the NSS or NKS trait can be used as an important criterion for accurately selecting varieties that may increase yield.

## 4. Discussion

One of the main goals of wheat breeding programs is to produce new varieties that are tolerant to salinity stress. The effective screening of salt-tolerant wheat genotypes that are appropriate to poor irrigation water quality and/or land salinization is greatly needed [43,55]. The increased rate of land salinization continues to harm yield depending on exposure duration and salt concentration, although low echelons of salinity may not necessarily reduce yield until a certain salinity “threshold” is reached [55]. Salt stress tolerance is a complicated trait, which, due to several interdependences, involves a comprehensive set of physiologic, biochemical, and molecular contributions in plants. Excessive salinity within growing media may cause several metabolic obstacles that prevent normal growth, such as increased osmotic effects, ion toxicity, and oxidative damage, and reduced effectivity of CO_2_ gas exchange, water status, and carbon metabolism as well as metabolic derangements [8,48,56]. Moreover, maintaining an optimal balance between Na^+^ and K^+^ is a crucial aspect of salt tolerance, which may also become an obstacle if uncontrolled [57]. To maintain the optimal balance between Na^+^ and K^+^ in metabolically active tissues, plants need to undertake energy-consuming operations to exclude and/or marginalize the role of Na^+^ combined with better uptake of K^+^, which is often done through the active transport of ions against a concentration gradient, thereby using a substantial amount of ATP in the process [58].

We evaluated five genotypes under salinity stress conditions (four DHLs and a check cultivar), which were used in our previous studies and have proven their tolerance to salt stress at the seedling stage (28 d) [5] and vegetative stage (43 d) [8], to measure their sustainability until harvest. The five salt-tolerant wheat genotypes (Sakha93, DHL2, DHL5, DHL21, and DHL26) showed different degrees of salt tolerance when subjected to 150 mM NaCl (Figure 1A). Four genotypes (Sakha93, DHL5, DHL21, and DHL26) started to show phenotypic variations just at the floret initiation stage, and DHL2 at the seedling stage showed salt sensitivity but quickly recovered to become more tolerant (Figure 1 and Figure S2). Our findings showed significant differences (*p* < 0.001) between salinity levels and genotypes, as well as their interaction with the tested traits for five wheat genotypes. Under salinity stress conditions, all parameters decreased in each of the five genotypes, except for CT and EC initial that showed the greatest decline in the check cultivar (Sakha-93). It was noted that the decreased parametric values was primarily a result of the imbalance of cell growth and division due to Na^+^ accumulation [45]. The deficit in plant growth is the first visible sign of salinity toxicity due to decreased hydraulic conductivity in plants [5,58].

CT is a useful indicator of water status in plants [8], and its reduction vis-à-vis surrounding air temperature implicates the efficiency of transpiration as a leaf-cooling response to stress. In the present study, check cultivar (Sakha-93) showed the highest CT value (Figure S1). Genetic differences among genotypes in terms of CT may be due to the ability of plant to move water across the vascular system, differences in stomata aperture that drives transpiration, root biomass and depth, metabolism, and source sink balance [59]. As such, CT is considered relevant to physiological traits, which is considered as a low-cost alternative measure for selecting salt-tolerant genotypes [35,60,61]. However, in plant breeding and selection of salt stress-tolerant varieties, the interest is in finding genotypes that maintain transpiration, gas exchange, and lower CT, compared with other genotypes under the same conditions [62], which was reflected in DHL2 in the present study (Figure S1).

Salt-tolerant varieties were proven to remain alive but accompanied by great deficiencies in productivity at an NaCl concentration of 120 mM [36,37]. The check cultivar (Sakha93, Figure 1A) died under saline irrigation treatment (150 mM NaCl) at the beginning of the flowering stage (critical age wherein optimal nutrition is most needed), although in several studies, it is considered as one of the tolerant genotypes at different stages of its lifespan [38,63,64,65]. El-Hendawy, et al. [64] found that the productivity of the Sakha93 cultivar decreased by 50% when irrigated with artificial saline water containing 7.02 g NaCl L^−1^ (120 mM), without dying, compared with the control group that was continuously irrigated with fresh water under field growing conditions. The incontinuity in these findings is due to the increased salt concentration (from 120 to 150 mM), wherein the plants were adversely affected by Na^+^ ion toxicity.

Considering the measurements in the entire research, DHL2 phenotypically manifested high salt tolerance, which had better value in most measurements, followed by DHL21 and DHL26. Despite the reduced Chl content in the leaf area (0.38 cm^2^) of DHL2 relative to other DHLs in control, there was, interestingly no significant reduction in Chl content in DHL2 under salt stress conditions (Figure 2). Furthermore, it showed better value in total pigment content (a, b, and total) under salinity condition, mainly owing to the higher FLA, compared with other DHLs (Figure 3). Most of all, Na^+^ and K^+^ concentrations were higher in DHL2 than other genotypes, but Na^+^/K^+^ ratio was very low relative to Sakha93 (Figure 7). Therefore, DHL2 may have used Na^+^ as an osmoticum to tolerate salt and osmotic stress, having demonstrated superior ability for tissue-tolerance. This could play a role in the great extent of carbohydrate storage through photosynthesis for as long as possible [48,66]. The inability of plants to avoid salt from reaching toxic levels in transpiring leaves may obstruct photosynthesis.

Maintaining a low cytosolic Na^+^/K^+^ ratio and detoxifying ROS are two fundamental mechanisms that enable plants to tolerate salinity [5]. Plant catalase proteins are implicated in H_2_O_2_ scavenging [8], and Na^+^ transporters NHX1 and HKT1 are two key actors in Na^+^ homeostasis [48]. In the present study, qPCR analysis revealed that DHL21 and DHL5 exhibited higher expression levels of the three stress-related genes (TaNHX1, TaHKT1, and TaCAT1) compared with DHL2 and Sakha93 under salt stress conditions (Figure 10). These findings were consistent with the low Na^+^/K^+^ ratio found in DHL21 and DHL5 genotypes, which exhibited tolerant phenotypes under salt stress conditions. Recently, Sharbatkhari, et al. [67] reported a high transcript rate of the NHX1 gene in both the leaves and roots of the salt-tolerant wheat genotype Kharchia Local that was subjected to salt stress conditions. Furthermore, Almeida, et al. [18] confirmed that the downregulation of the TaHKT 1;5 gene in transgenic bread wheat lines enhanced Na^+^ accumulation in the leaves and led to increased salt stress susceptibility. Likewise, several earlier reports have demonstrated that tolerant wheat genotypes exhibited increased activities of antioxidant enzymes, which is associated with the upregulation of corresponding genes [19,22,68,69]. Overall, the salt-tolerant phenotype of the DHL21 and DHL5 appeared to be due to the efficiency of maintaining ionic homeostasis and high ability to detoxify hydrogen peroxide (Figure 10).

The GY loss in DHL21 (52%) was more severe than that in DHL2, DHL5, and DHL26 (~38%) (Figure 9). This implied that photosynthesis helped the plants to fill the grain compartments under stress conditions [31]. It eventually led to better yield preservation in salt-tolerant cultivars, although salt stress significantly decreased yield and yield components for both salt-sensitive and -tolerant genotypes [34,70]. Salt-tolerant genotypes can beat the adverse effects of salinity by combining ion-exclusion and tissue-tolerance strategies, and understanding their joint contribution to salt tolerance can be of immense interest for developing salt-tolerant cultivars [71,72]. Understanding how the measured interpretive traits positively or negatively influence the GY, as well as their contributions under salinity stress, can be a powerful tool for plant breeders for decision-making on breeding strategy programs, especially if they used methods that are easily measurable, the least expensive, quick, and stable [5,73]. The correlation between traits described the association between traits under salinity stress conditions and identification of related interpretive traits to increase yield [48]. In the present study, the nine measured interpretive traits [LWC, RWC, Chl a (0.38 cm^2^), Chl b (FLA), Chl a + b (FLA), EC final, NSS, NKS, and KA] could be unbiased parameters for assessing GY under salinity stress conditions due to their significant contributions to GY (Figure 11). However, depending on simple correlations, without regarding the interactions between GY and the related interpretive traits, can mislead plant breeders in achieving the major objective of the program [45]. Therefore, we used multivariate analyses methods (PCA, MLR models, and path coefficient analysis), which were used in several studies [5,43,74].

PCA has been used to define the most crucial traits using the first two components, which explained 70.78% of the overall variation of all traits. Accordingly, 28 out of 32 measured traits were loaded onto PC1 and PC2 (with scores > 0.348), and 28 traits were used as an efficient screening criterion (Table S2, Figure 12). Generally, the acute angle between the vector of the GY trait and the measurements of leaf water status (except CT), chlorophyll content [except Chl a (0.38 cm^2^)], membrane injury (EC initial), photosynthetic, flag leaf, shoot K^+^ ion concentration, and yield components (except KLWR) was acute (less than 90°), indicating that these traits were strongly and positively associated with GY and were directly and/or indirectly estimated (Figure 12). MLR models and path coefficient analysis were used for all traits as interpretive variables; hence, the dependent variable allowed us to identify the relationships among traits under salinity stress conditions.

The stepwise and forward regression models involved merging as few variables as appropriate because each unrelated repressor diminishes the precision of the coefficients and expected values. Additionally, the variables that were added in the process further complicated our data collection and model safeguarding strategies. The three models of regression have a crucial coefficient of determination (R^2^) of 0.93 from 1.000. In the present study, the two interpretive variables, NSS and/or NKS, served as unbiased traits for assessing wheat DHLs under salinity stress conditions given their contribution to and direct impact on GY as a dependent variable (Table 1). The crucial contribution of NSS and/or NKS to GY supported their importance as selection criteria in wheat breeding programs. Therefore, they may be used as criteria for screening and selection to evaluate the best genotypes that can survive under salt stress, and in particular NSS, at an early phase after the flowering stage, but only after removing the MSI and Chl a (0.38 cm^2^) traits due to their weak contributions [75,76,77].

## 5. Conclusions

Our results indicate that the use of rapid, convenient, and low-cost methods as a preliminary assessment can help breeders in assessing key plant traits relevant to stress tolerance that are necessary under fully controlled conditions. The genotypes DHL2 and DHL21 showed competitiveness and high performance under saline stress conditions. Therefore, these two most salt-tolerant genotypes can be useful as genetic resources for future breeding programs that would further the possibility of generating a new salt-tolerant genetic source of wheat through hybridization of the desired genotypes, which is expands the genetic base for salt tolerance breeding in wheat. Nevertheless, further investigation is needed under greenhouse conditions in order to confirm the dose-dependent tolerance of Sakha93 and, in the field conditions using 150 mM NaCl, to confirm the better tolerance performance of the genotypes DHL2 and DHL21.

## Figures and Tables

**Table 1:** Summary of multiple linear regression analysis (best model, stepwise, and forward) and path analysis (direct and indirect effects) for grain yield (dependent variable) with 28 yield-related traits (independent variables) for nine treatments.

No. of Variables	Source	Without Deleting						After Deletion of NKS						After Deletion of NSS					
		Best Model		Stepwise		Forward		Best Model		Stepwise		Forward		Best Model		Stepwise		Forward	
	Independent Variables	R^2^	Direct Effect	R^2^	Direct Effect	R^2^	Direct Effect	R^2^	Direct Effect	R^2^	Direct Effect	R^2^	Direct Effect	R^2^	Direct Effect	R^2^	Direct Effect	R^2^	Direct Effect
2	MSI/NKS	0.94	0.75											0.94	0.75	0.94	0.75	0.94	0.75
2	Chl a (0.38 cm^−2^)/NSS			0.93	0.78	0.93	0.78	0.93	0.75	0.93	0.78	0.93	0.78						
3	NKS/HKW/KL	0.97												0.97					
3	Chl a (0.38 cm^−2^)/FLA/NSS							0.97											
4	Chl a (0.38 cm^−2^)/Chl a + b(FLA)/NSS/KW	0.99						0.99											
4	FLA/E/NKS/Na^+^													0.99					
5	RWC/Chl a (0.38 cm^−2^)/Chl b(FLA)/NSS/KW	1.00						1.00											
5	LWC/FLA/Gs/NKS/Na^+^													1.00					
1	Chl a (0.38 cm^−2^)			0.08	0.09	0.08	0.09		0.09	0.08	0.09	0.08	0.09						
1	NKS		0.62												0.62	0.82	0.62	0.82	0.62
1	NSS			0.85	0.69	0.85	0.69		0.66	0.85	0.69	0.85	0.69						
1	MSI		0.13												0.13	0.12	0.13	0.12	0.13
	Total indirect effect		0.19		0.15		0.15		0.18		0.15		0.15		0.19		0.19		0.19
	Total R^2^		0.94		0.93		0.93		0.93		0.93		0.93		0.94		0.94		0.94
	Residual		0.24		0.26		0.26		0.26		0.26		0.26		0.24		0.24		0.24

## Data Availability

All data is contained within the article or supplementary material.

## Supplementary Materials

The following are available online at https://www.mdpi.com/2079-7737/10/1/56/s1, Table S1: List of primers used in Real-Time qPCR; Table S2: Principal component analysis of five wheat genotypes, eigenvalues, proportion, and cumulative variance for the first six components for the measured traits of nine treatments; Figures S1–S4.
