# Supplementary material for: Agro-Physiologic Responses and Stress-Related Gene Expression of Four Doubled Haploid Wheat Lines under Salinity Stress Conditions
Source: Biology (Basel). 2021 Jan 14;10(1):56. doi: 10.3390/biology10010056 (PMC7828821; doi:10.3390/biology10010056)
Supplement: Supplementary file 1 [file biology-10-00056-s001.pdf]

biology

Supplementary material

Agro-Physiologic Responses and Stress-Related Gene Expression of Four Doubled Haploid Wheat Lines Under Salinity Stress Conditions

Ibrahim Al-Ashkar, Walid Ben Romdhane, Rania A. El-Said, Abdelhalim Ghazy, Kotb Attia and Abdullah Al-Doss

Table 1. List of primers used in Real-Time qPCR.

Primer Name	Sequence 5′–3′
TaCAT1-F	TCTCTCGGCCAGAAGCTCG
TaCAT1-R	AGGGAAGAACTTGGACGGC
TaNHX1-F	TGACGGAGGCAGAAGACCG
TaNHX1-R	CCCAAAACTCTACACAGCGT
TaHKT1 ;5-F	TTGGTATAACTGTCGCTAGTG
TaHKT1 ;5-R	TGGGGAACTCATGTGGTTAC
TaAct-F	ATACAGTGTCTGGATCGGTG
TaAct-R	TCATACAGCAGGCAAGCACC

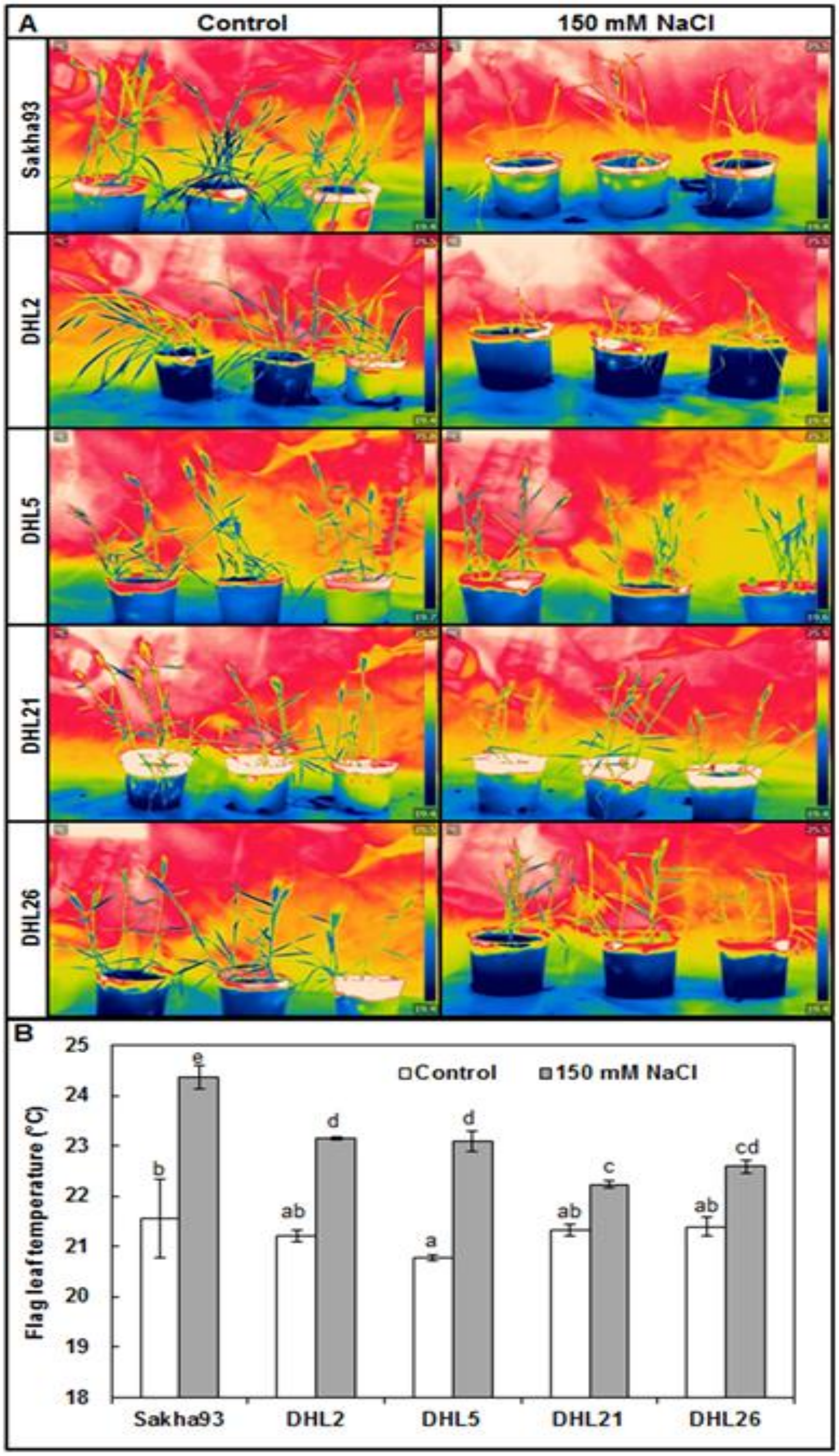

Figure S1. Thermal images of five wheat genotypes subjected to irrigation water with and without 150 mM NaCl taken at the early floret initiation stage (A). Changes in flag leaf canopy temperature in response to 150 mM NaCl in five wheat genotypes (B). Values are presented as the mean ± SE of at least three replications, and values sharing the same letter for each treatment × genotype combination are not significantly different ($P \leq 0.05$) according to Duncan's multiple range test.

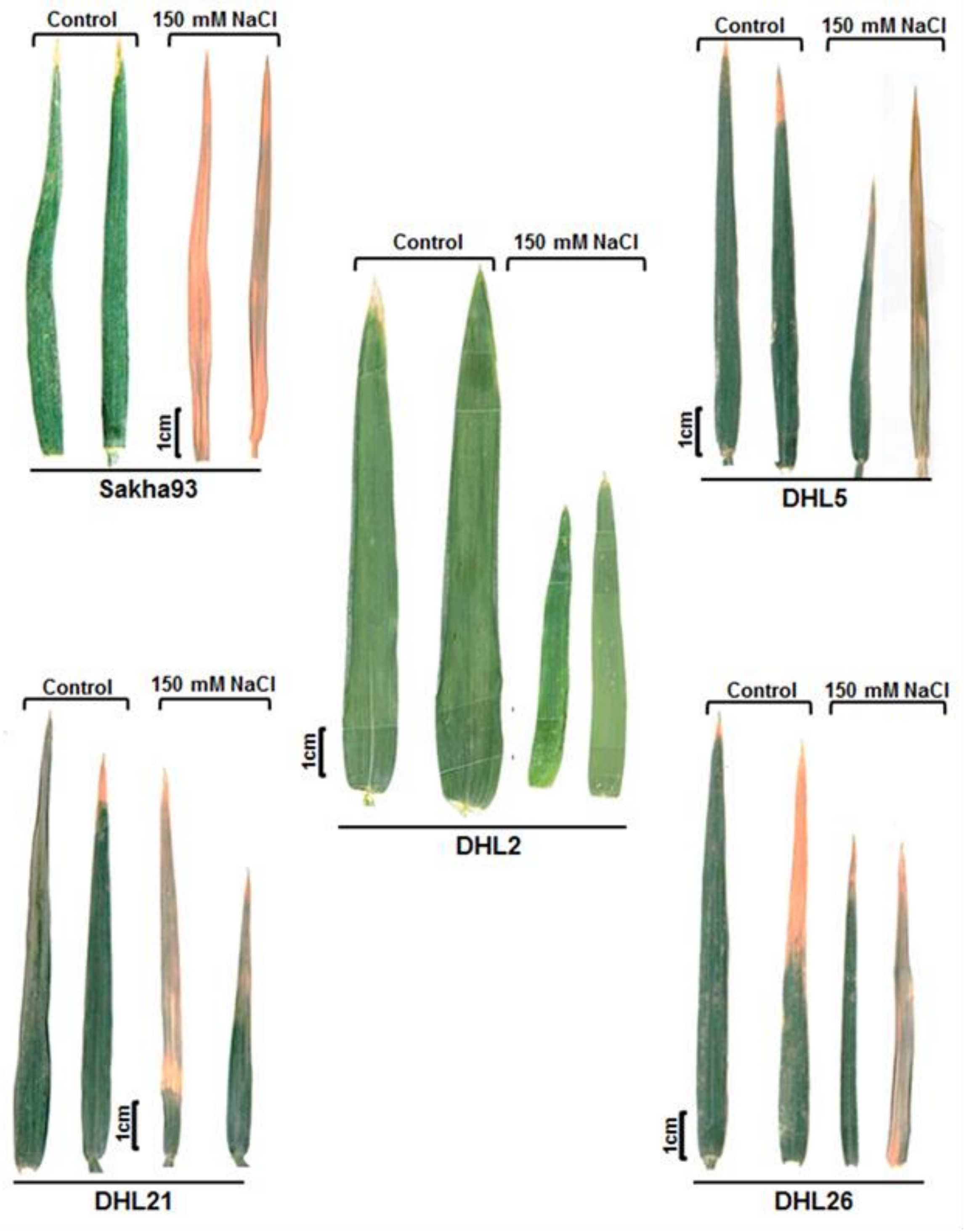

Figure S2. Flag leaf morphology of five wheat genotypes under control and salt stress conditions.

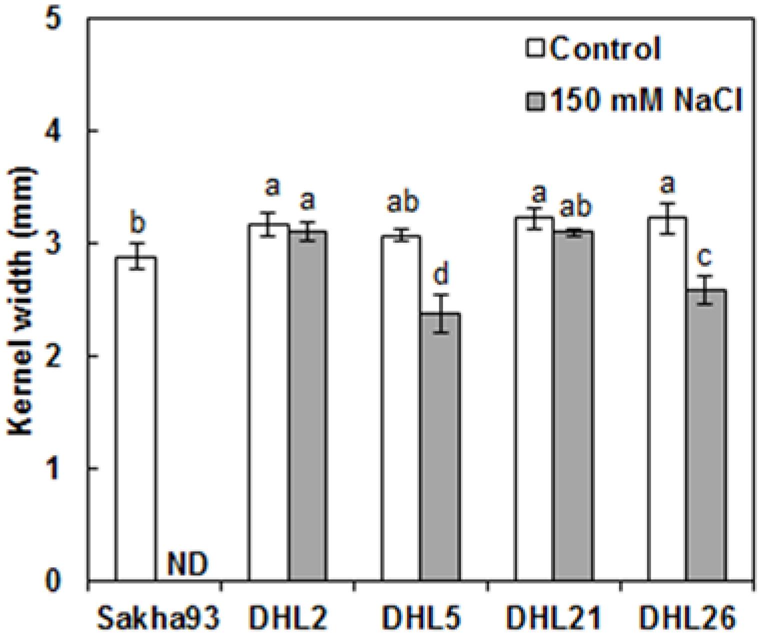

Figure S3. Kernel width of five wheat genotypes in response to salinity. Values are presented as the mean ± SE of at least ten replicates, and bars sharing the same letter are not significantly different (*P* ≤ 0.05)

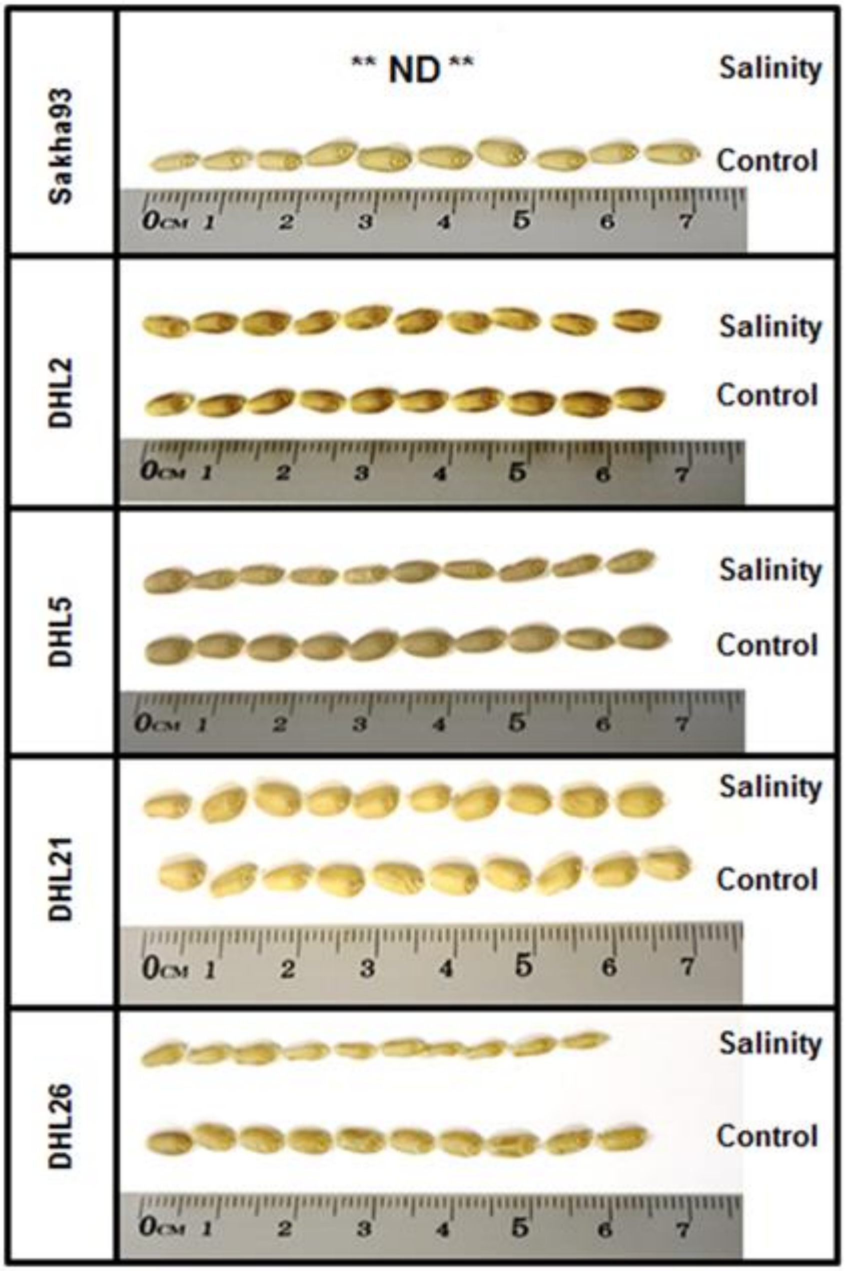

Figure S4. Kernel morphology of five wheat genotypes under control and salt stress conditions.

Table 2. Principal component analysis of five wheat genotypes, eigenvalues, proportion, and cumulative variance for the first six components for the measured traits of nine treatments.

Value	PC1	PC2	PC3	PC4	PC5	PC6
Eigenvalue	15.10	7.55	3.20	2.08	1.74	1.45
Variability (%)	47.19	23.60	9.99	6.49	5.44	4.52
Cumulative %	47.19	70.78	80.77	87.27	92.70	97.23
Component loading*						
LWC	**0.56**	0.09	0.00	0.15	0.03	0.11
RWC	**0.64**	0.09	0.01	0.00	0.04	0.14
CT	**0.45**	0.34	0.00	0.00	0.05	0.12
Chl a (0.38 cm^{-2})	0.02	**0.81**	0.00	0.01	0.06	0.00

Chl b (0.38 cm^{-2})	0.15	**0.73**	0.01	0.02	0.04	0.02
Chl a+b (0.38 cm^{-2})	0.03	**0.86**	0.00	0.02	0.05	0.01
Chl a (FLA)	**0.73**	0.20	0.03	0.00	0.03	0.00
Chl b (FLA)	**0.83**	0.10	0.03	0.00	0.03	0.00
Chl a+b (FLA)	**0.79**	0.14	0.03	0.00	0.03	0.00
EC initial	0.17	0.01	**0.71**	0.08	0.00	0.00
EC final	**0.75**	0.12	0.07	0.00	0.02	0.00
MSI	**0.74**	0.01	0.20	0.03	0.02	0.00
FLL	**0.63**	0.31	0.04	0.00	0.02	0.00
FLW	0.44	**0.51**	0.00	0.01	0.01	0.01
FLA	**0.52**	0.42	0.04	0.00	0.01	0.00
FL angel	0.09	0.14	0.04	0.08	0.08	**0.53**
Pn	**0.59**	0.04	0.00	0.00	0.33	0.03
Gs	**0.63**	0.02	0.16	0.16	0.00	0.02
Ci	0.22	0.06	0.10	**0.35**	0.25	0.01
E	**0.65**	0.03	0.09	0.15	0.00	0.05
SL	**0.68**	0.02	0.12	0.05	0.02	0.09
NSS	**0.61**	0.11	0.02	0.20	0.03	0.00
NKS	**0.42**	0.07	0.05	0.41	0.00	0.00
HKW	**0.46**	0.01	0.30	0.03	0.14	0.01
KW	**0.54**	0.01	0.41	0.00	0.02	0.01
KL	0.24	**0.53**	0.02	0.01	0.15	0.05
KA	**0.58**	0.28	0.09	0.00	0.03	0.01
KLWR	0.07	0.23	**0.47**	0.01	0.15	0.05
K^+	**0.67**	0.16	0.04	0.00	0.04	0.03
Na^+	0.02	**0.80**	0.10	0.00	0.00	0.02
Na^+/k^+	**0.50**	0.29	0.00	0.01	0.04	0.13
GY	**0.68**	0.01	0.00	0.28	0.00	0.01

* values ≥ 0.35 are presented in bold face and indicates traits important for PC definition; Leaf water content (LWC), relative water content (RWC), Chlorophyll content [a, b, and total (in cm-2 and total flag leaf area)], membrane stability index (MSI), flag leaf length (FLL), flag leaf width (FLW), flag leaf area (FLA), and flag leaf angle (FL angle), photosynthesis rate (Pn), transpiration rate (E), stomatal conductance (Gs), and intracellular CO2 concentration (Ci), spike length (SL) number of spikelets (NSS), number of kernels (NKS), 100-kernel weight (100-KW), , kernel length (KL), kernel width (KW), kernel area (KA), , kernel length–width ratio (KLWR) Na+ and K+ concentrations, K+/Na+ ratio and grain yield (GY).
